# Providing Antivenom Treatment Access to All Brazilian Amazon Indigenous Areas: ‘Every Life has Equal Value’

**DOI:** 10.3390/toxins12120772

**Published:** 2020-12-05

**Authors:** Wuelton Marcelo Monteiro, Altair Seabra de Farias, Fernando Val, Alexandre Vilhena Silva Neto, André Sachett, Marcus Lacerda, Vanderson Sampaio, Deugles Cardoso, Luiza Garnelo, João Ricardo Nickenig Vissoci, Jacqueline Sachett, Fan Hui Wen

**Affiliations:** 1 Department of Medicine and Nursing, School of Health Sciences, Amazonas State University, Manaus 69065-001, Amazonas, Brazil; wueltonmm@gmail.com (W.M.M.); asfarias@uea.edu.br (A.S.d.F.); ffaval@gmail.com (F.V.); jac.sachett@gmail.com (J.S.); 2 Department of Teaching and Research, Dr. Heitor Vieira Dourado Tropical Medicine Foundation, Manaus 69040-000, Amazonas, Brazil; alexandre.neto94@gmail.com (A.V.S.N.); andre.sachett@gmail.com (A.S.); marcuslacerda.br@gmail.com (M.L.); vandersons@gmail.com (V.S.); 3 Instituto Leônidas & Maria Deane, Fiocruz, Manaus 69057-070, Amazonas, Brazil; luiza.garnelo@fiocruz.br; 4 Technical Department, Amazonas Health Surveillance Foundation, Manaus 69093-018, Amazonas, Brazil; fvs.gerenciadezoonoses@gmail.com; 5 Division of Emergency Medicine, Department of Surgery and Duke Global Health Institute, Duke University, Durham, NC 27710, USA; jnv4@duke.edu; 6 Department of Teaching and Research, Alfredo da Matta Foundation, Manaus 69065-130, Amazonas, Brazil; 7 Bioindustrial Centre, Butantan Institute, Butantã 05503-900, São Paulo, Brazil

**Keywords:** snakebite, antivenom, indigenous groups, health service, health decentralization

## Abstract

Snakebites are more frequent in the Brazilian Amazon than in other parts of Brazil, representing a high cost for the health system since antivenoms are only available through medical prescription from central municipal hospitals in most cases. The need for a cold chain and physicians usually restricts access to the only effective treatment of a snakebite, the antivenom. The complex topography of the rivers contributes to delays in treatment, and consequently increases the risk of severe complications, chronic sequelae and death. Thus, decentralization of antivenom treatment to primary healthcare facilities in the interior would increase access by indigenous population groups to proper healthcare. To standardize and evaluate the decentralization to low complexity indigenous healthcare units, we suggest the (i) development and validation of standardized operational procedures, (ii) training of professionals in the validated protocol in a referral health unit, (iii) implementation of the protocol in an indigenous healthcare unit, (iv) assessment of perceptions towards and acceptability of the protocol, and (v) estimation of the impact of the protocol’s implementation. We expect that antivenom decentralization would shorten the time between diagnosis and treatment and, as such, improve the prognosis of snakebites. As health cosmology among indigenous populations has an important role in maintaining their way of life, the introduction of a new therapeutic strategy to their customs must take into account the beliefs of these peoples. Thus, antivenom administration would be inserted as a crucial therapeutic tool in a world of diverse social, natural and supernatural representations. The information presented here also serves as a basis to advocate for support and promotion of health policy initiatives focused on evidence-based care in snakebite management.

## 1. Introduction

Much of the Amazon rainforest is contained in Brazil (60%) and, as such, it comprises the largest and most diverse tract of tropical rainforest in the world. In 2019, there were 30,482 snakebite cases reported in Brazil, 13,601 of which were in the Brazilian Amazon Region. These data portray the uneven distribution of the problem across the country. The Brazilian Amazon region accounts for only 8.7% of Brazil’s total population, yet it suffers 44.6% of reported snakebite envenomations; an incidence that is five times higher than in the rest of the country [1]. In this region, the annual costs associated with snakebites represent over US$ ~8 million, of which US$ ~4.5 million is due to lost productivity, secondary to premature death and morbidity [2].

Antivenoms are the only effective treatment against the effects of venomous snakebites. Thus, favoring access to health units with appropriate antivenoms is crucial to avoid complications, sequelae and death [3]. In Brazil, the acquisition of antivenoms (AV) is the exclusive responsibility of the Brazilian Ministry of Health [3], which, in turn, distributes them to the states. State governments then distribute them to the municipalities. The replacement of antivenom stocks depends on snakebite notifications. As a result, decision making regarding the regional distribution of AV hinges on (a) the number of cases detected by the official surveillance system; (b) the conditions for cold storage; and (c) the availability of hospital facilities and proper medical supervision. Health facilities that are well equipped and staffed with trained personnel are vital to mitigate possibilities of early adverse reactions to AVs, particularly the risk of anaphylactic reactions since the product contains equine proteins [4].

Snakebites are medical emergencies that typically require the early use of antivenom, preferably within the first six hours [5]. However, for many vulnerable groups, such as the indigenous tribal communities, it may take days to obtain adequate health care, thus missing out on the crucial treatment window. Subsequently, with this delay, the antivenom is no longer useful in reversing the effects of envenomation [5,6]. The precise number of patients who remain deprived of antivenom treatment remains unknown, because snakebites burden in remote rural areas is under-reported [7,8,9]. Detailed surveys are essential in order to clarify the extent to which individuals and families in the indigenous territories within the Amazon region are deprived of this crucial intervention. In the real-life scenario, the availability and accessibility to the antivenom are very uneven across the Brazilian territory.

Additionally, cultural factors that are firmly rooted among the riverine and indigenous populations in the Brazilian Amazon can also contribute to an individuals’ decision not to seek proper medical care and, instead, opt for treatment using traditional medicine [10,11]. Thus, the current policy criteria for antivenom distribution in Brazil needs reviewing, since it deprives a considerable portion of the population living in the Brazilian Amazon from promptly accessing antivenom treatment.

This study comprehensively reviews the literature in regards to snakebites that occur among indigenous populations in the Brazilian Amazon and discusses the implementation of decentralization of antivenom therapy to low complexity health units within the aforementioned region.

## 2. Indigenous Populations in the Brazilian Amazon

**Figure 1:**
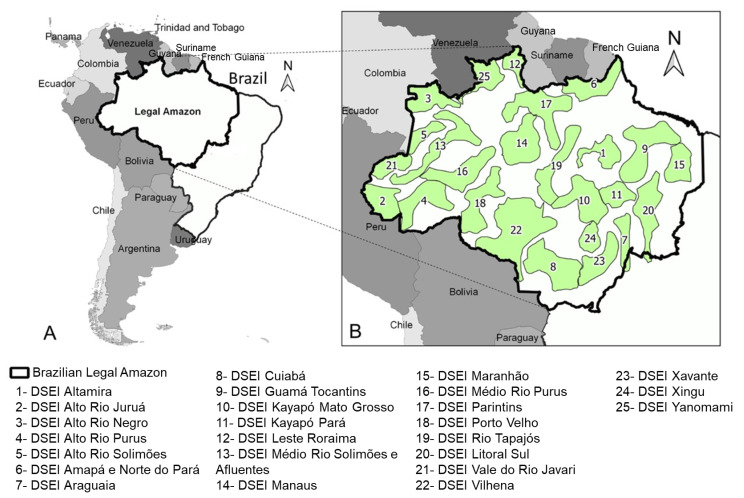
The Legal Amazon within the Brazilian territory (**A**) and location of indigenous health districts in Brazil in the Brazilian Amazon (**B**).

The Brazilian Amazon covers an area of 5,217,423 km^2^ and accounts for 61% of Brazil’s national territory (Figure 1A). It is a large territorial extension with a low population density. The Brazilian government recognizes 690 indigenous territories that cover over 13% of the Brazilian territory and almost all of these territories are in the Amazon. About 60% of the total of 896,917 self-declared Brazilian indians (0.4% of Brazil’s population) live in the Brazilian Amazon, and belong to more than 305 indigenous peoples who speak 274 languages [12]. The Yanomami ethnic group occupies the largest territory (9.4 million hectares) in the Amazon. This indigenous group with a population of almost 27,000 lives in relative isolation in the northern Amazon. At 53,000 strong, the Tikuna are the most populous indigenous community in the Brazilian Amazon [12]. Most of these indigenous groups live in the forests and survive by hunting, gathering and fishing. They also grow crops for food, especially cassava, and use plants for their medicines. In addition, they also use locally sourced vegetation for housing materials and everyday objects and accessories. Indigenous populations are classified as villagers (*aldeados*, in Portuguese) or nonvillagers (*não-aldeados*). The villagers inhabit territories, traditionally owned and occupied by their ancestors, demarcated and protected by the Brazilian Constitution. Nonvillagers, on the other hand, are individuals that identify themselves as indigenous but inhabit any space outside these demarcated lands [13].

### 2.1. Indigenous Health Care

Therapeutic practices vary among the various ethnic groups. Appointed individuals within the community, such as shamans, those with knowledge of medicinal plants and animal-based medicines, often provide the treatment in these settings [14]. The shamanistic spiritual rituals serve as an intermediary between culture and religion, and include an extensive collection of natural “antidotes”, cultivated in or extracted from the jungle by indigenous families [15,16]. In Brazil, indigenous health agents and a team composed of nurses, physicians, dentists and other healthcare professionals who are employed by the Brazilian government provide health care services to these communities. Indigenous people also receive crucial health care services from military personnel, missionaries and church-based groups and other nongovernmental organizations. Self-medication with commercial medicines is another increasingly common form of therapeutic intervention used among almost all indigenous ethnic groups. All of these “western” therapeutic resources and interventions are complementary and do not replace traditional medicine [17].

The main framework for public policies regarding indigenous health in Brazil was established through the Federal Constitution (1988), which included the Indigenous Health Care Subsystem within the scope of the Unified Health System (SUS). The organizational model is based on Special Indigenous Health Districts (DSEIs) [18]. There is also a Special Secretariat for Indigenous Health within the Ministry of Health that grants administrative and budgetary autonomy to the DSEIs [19]. The DSEI is a service organization model that is charged with providing a set of primary health care services to Brazil’s indigenous communities within their ethnocultural space [18]. Of the 34 DSEIs in Brazil, 25 are in the Brazilian Amazon and serve a population of 465,253 indigenous villagers (Figure 1B) [19].

**Figure 2:**
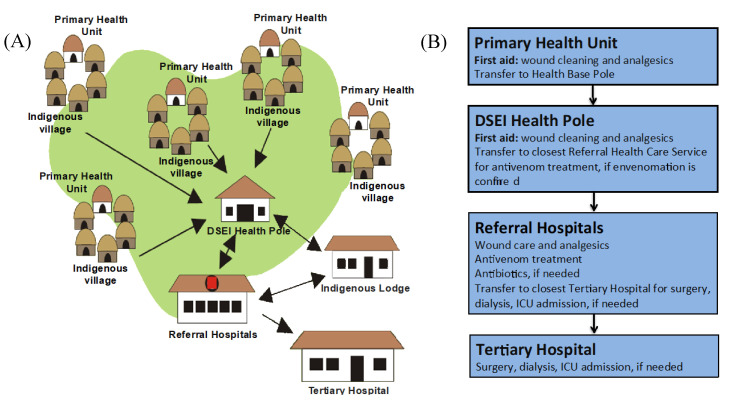
Structure of the indigenous health districts (**A**) and flow of care for envenomations (**B**). Removing the patient from the reservation territory is considered a critical event and can cause anxiety for these people, especially when they need complex health care procedures. For some communities, surgical procedures are considered an aggression that will trigger an illness. The Sanumá-Yanomami shamans, for example, regard surgical cuts as a possibility of poison being inoculated into their tribesman’s blood, which generates “old blood” and is capable of causing harm to the patient or a bad omen [19]. Furthermore, removing the patient to the city requires special protection with charms, spells and food. They believe that foods produced in different environments and by other people can cause their transformation into other beings or hinder health recovery [20,21].

The first referral service for the Multidisciplinary Indigenous Health Teams is a primary health unit, which is sited in the indigenous village. If the patient’s needs require specialized attention, they receive a referral to the nearest easily accessible DSEI Health Pole. When in need of advanced or specialized medical services, antivenom treatment included, the indigenous patient is referred by the referral hospital in the nearest municipal headquarters (Figure 2).

### 2.2. Cosmology of Health and Snakebites among Indigenous Populations

For different Amerindian indigenous populations, diseases are not necessarily the result of etiological agents causing structural or functional anomalies in a biological system [22]. Instead, the appearance of disease reflects an individual’s set of actions, his activities in the society and the natural environment. A health problem is not considered outside of personal and social uniqueness, personal and historical circumstances or representations of the natural world, but the forces that govern them and the relationships between the human, natural and supernatural world. Interventions by human agents, spirits, animals and deities are believed to cause diseases in the community, with resultant consequences of a socially deviant behavior or by violating the cultural rules [17]. Likewise, preventive measures and health care are linked to interaction with the environment with considerable symbolism and representations [14]. Indigenous people not only live with nature and biodiversity, where they and other elements are diffuse, but they also name and classify living species using a method of naming and categorizing that is based on the natural world and their cultural domain [23].

For the indigenous people of the Rio Negro, in general, snakebite *status* implies severe dietary restrictions, as they understand that the consumption of certain fish and game animals is antagonistic to the body and spirit, and this worsens the disease. The rules specifically prohibit snakebite victims from consuming any fish capable of biting and stinging, or having physical characteristics (smooth, slimy, scales-free, venomous) resembling those of snakes. Victims of snakebites and their immediate families are obliged to comply very strictly to dietary requirements, including restriction to the consumption of mammals, in particular beef and game animals. They believe that it may increase bite-induced edema and necrosis. Therefore, they refuse going to city-based hospitals, as they know that while there following their traditional diets will be a problem and they will be coerced into eating animal meat [22], contrary to their customs.

In general, indigenous people do not understand that snakebites occur by chance, but because the individual has left himself vulnerable to being attacked, has disregarded existing dietary rules or because they have behaved inappropriately [24]. The Sanumá-Yanomami, for example, obviously know that the cause of death from a snakebite is the venom inoculated by the animal, but what interests them most is why the victim was bitten [20]. Thus, a snakebite is understood not as a set of signs and symptoms, but as spiritual aggression that is indiscernible to most people [20]. As a preventive measure, there are dietary restrictions and rituals that, if disobeyed, can leave the person vulnerable to snakebites. Also, there are societal conditions (beliefs) that are to be taken into account when attending a snakebite victim, such as the prohibition of care from pregnant or menstruating female caregivers. In regards to food, the Baniwa recognize that, to a greater or lesser extent, fish share the ability to bite with snakes, and possess and release toxic substances.

## 3. Snakebites in Amerindian Populations in the Amazon

**Figure 3:**
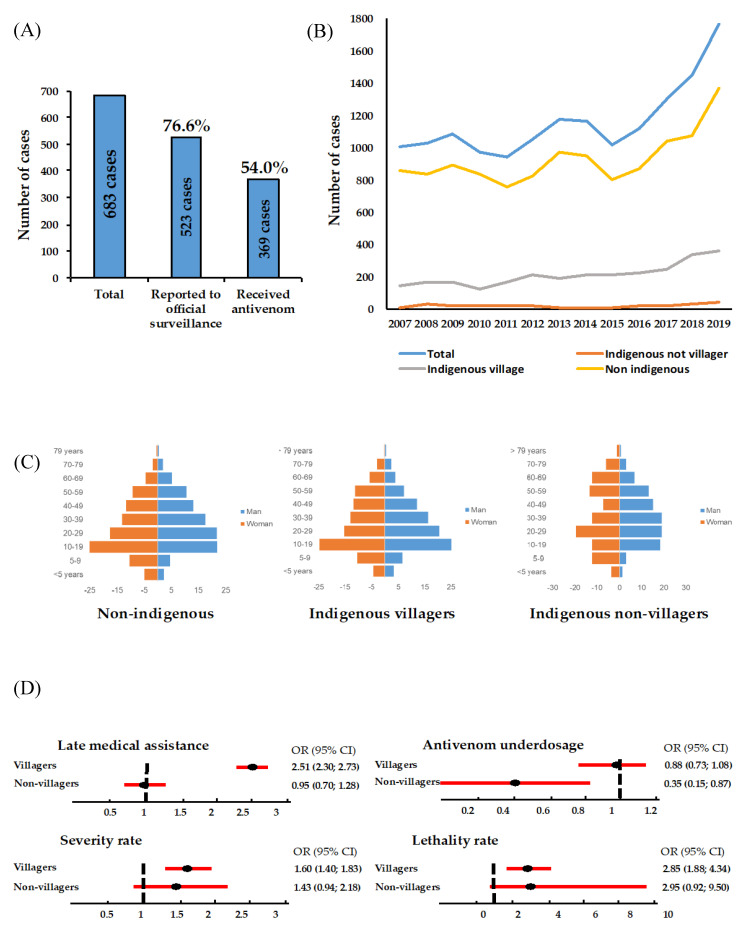
Frequency of under-reporting and antivenom treatment in indigenous groups living in six different DSEIs in the state of Amazonas (**A**), and comparison between temporal trends (**B**), age pyramids (**C**), frequencies of late medical assistance and antivenom underdosage, and lethality and severity rates (**D**) between nonindigenous populations, indigenous populations living in DSEIs (villagers) and indigenous nonvillagers, bitten by *Bothrops* snakes, in the state of Amazonas, Western Brazilian Amazon.

In the Amazon, distribution of the snakebite burden is disproportional among indigenous and nonindigenous populations. In an investigation conducted by the Amazonas State Health Surveillance Foundation (FVS-AM), 683 snakebites were counted in six DSEIs of this state. Of these, 523 (76.6%) were notified to the official epidemiological surveillance system and only 369 cases (54.0%) received antivenom treatment (Figure 3A). In 2019, the state of Amazonas recorded a prevalence of 44.2 cases/100,000 inhabitants among nonindigenous people, 72.2/100,000 in indigenous nonvillagers and 333.5/100,000 in indigenous villagers. Thus, despite the assumption that there is greater under-reporting of cases among the indigenous people than among nonindigenous people, the prevalence is at least 7.5 times higher according to the official surveillance system. If we take the case of the indigenous lands in the Upper Rio Negro as an example, the analysis of data from the Federation of Indigenous Organizations of Rio Negro (FOIRN) is explanatory. From 2017 to 2018, FOIRN conducted a participatory census using a wide range of interviews of residents of nine demarcated indigenous lands, which included 307 villages and 3523 indigenous families. The survey revealed 260 snakebites reported by respondents in the 12 months prior to the survey. Thus, indigenous people’s way of life involves daily interaction with snakes, and thus increases the risk of snakebites. The same interviewees recalled, for the same period, only 315 medical visits, for all health complaints. Such data express the point of view of indigenous users and, for them, snakebites are a relevant health problem, *pari passu* to precarious medical aid [25].

Despite sparse observations indicating that snakebites among the indigenous people may be more serious and lethal, few researchers have devoted themselves to understanding these differences in greater depth. Figure 3B, illustrates an increasing trend in the number of *Bothrops* spp. snakebite cases from 2007 to 2019, in the state of Amazonas, Western Brazilian Amazon. This increase in numbers was greatest among the indigenous villagers; while this population had an increase of 160%, nonindigenous people registered a 60% increase in the same period. This increase can be attributed to not only the actual increase in the number of snakebites, but also to an improvement in the DSEIs’ surveillance system.

Snakebites caused by *Bothrops* spp. are the most common in the Amazon, accounting for ~90% of the recorded cases [26]. Other native Brazilian species from the *Brothrops* genus, such as *B. jararaca*, *B. jararacussu*, *B. moojeni*, *B. brazili* and *Bothrocophias hyoprora*, are also involved in human snakebites in the region. Snakes from other species, such as *Lachesis muta*, *Crotalus durissus*, *Micrurus hemprichii* and *Micrurus lemniscatus*, have also been involved in human snakebites [4]. Figure 3C shows the sex and age distribution of *Bothrops* spp. snakebite patients among the nonindigenous populations, indigenous populations living in DSEIs (villagers) and indigenous nonvillagers, in the state of Amazonas. What stands out in all the three groups is that the snakebite victims are predominantly adult men. This indicates that this part of the population most likely carry out activities that make them more vulnerable to encounters with venomous snakes. Figure 3D shows a comparison between the population groups of risks in some clinically relevant outcomes. This analysis revealed that the frequency of delayed medical care, lethality, and severity was higher in indigenous villagers than in nonindigenous people and indigenous nonvillagers. Antivenom underdosing was also higher in indigenous nonvillagers. This suggests that, in addition to difficulties in accessing hospitals with stocks of the necessary antivenom, the quality of assistance the indigenous population needs was not adequate and requires optimizing. Indeed, the lethality rate from *Bothrops* spp. snakebites was significantly higher in indigenous nonvillagers (1.41%); and for indigenous villagers (1.40%) compared to nonindigenous populations (0.53%).

A probable explanation for these findings, although simplistic, is late medical attendance to the patient due to the long journeys from the villages to the hospital, the great distances covered to reach the primary healthcare units, along with their limited resolution. These snakebites often occur at home, in the jungle and in subsistence plantations where these victims work as farm laborers. The sites where these accidents occur more often than not are far from the nearest primary healthcare unit. To understand this, it is, however, necessary to consider the main challenges with cultural, social and economic disparities between cultures. It is believed that such aspects make it even more difficult for patients to travel from the place of residence or the area where the snake bite occurred, to a health service capable of handling the case adequately. Understanding and incorporating crucial factors, such as the healthcare providers’ knowledge of venomous snake species in the region and toxicological symptoms in snakebite patients, would support the choice of appropriate treatment programs and strategies to be used. Strategies that guarantee this affected population access to treatment services at an opportune time provide a link between adherence to appropriate treatment of the snakebite victims, and proper training of health professionals that deal with indigenous populations are key to providing the best care to members of the indigenous communities.

## 4. Current Logistics for Antivenom Delivery and Outcomes

In Brazil, in order to understand the distribution network of antivenom used to treat snakebites, one has to go back to the 1970s. In 1973, an important milestone was achieved by the institutionalization of the National Immunization Program (NIP), which in addition to promoting the control of vaccine-preventable diseases, was also responsible for improving public instruments for assessing the quality of immunobiologicals [27]. The National Institute for Quality Control in Health (INCQS) was created in 1981, and it soon detected some contamination of imported vaccine batches used in government campaigns. INCQS also detected important flaws and deficiencies in other biological products, including antivenoms. At the time, there was also a technological gap in the national laboratories.

In the 1980s, Brazil’s economic fragility and its dependence on foreign industries became evident when Syntex do Brasil decided to suspend the manufacture of vaccines and snake antivenoms, casting the country into a serious antivenom and vaccine supply crisis. At the time, Syntex do Brasil was a multinational pharmaceutical company that was providing the majority of vaccines and antivenoms being used in Brazil. State-owned national laboratories represented by Instituto Butantan, Instituto Vital Brazil and Fundação Ezequiel Dias had a small market share in the supply of snake antivenoms at the time and were not in a position to meet national demand. Consequently, withdrawal by the multinational company resulted in a shortage of antivenom in early 1985, a crisis marked by an increasing number of amputations and deaths from snakebites in the country.

Given the seriousness of the situation, the Ministry of Health initiated a program to improve the industrial infrastructure and production capacity by these state laboratories [3]. Parallel to the investment in antivenom production, the National Snakebite Program was created in 1986 and soon became the National Program for the Control of Accidents by Venomous Animals. One of the core functions of the program was the implementation of a case surveillance system and the creation of a network of accredited healthcare services for antivenom treatment. The Ministry of Health centralized the purchase of snake antivenom, while the National Immunization Program (NIP) was tasked with its distribution to the states, along with vaccines. As a result, commercial sale of the vital immunobiological commodity ceased to exist, with antivenoms made available at no cost to the patients in the healthcare services of a unit chosen as a reference center for antivenom treatment.

Development of criteria to determine the supply of antivenom to certain health services was influenced by: (i) local epidemiological relevance; (ii) training of health professionals regarding the diagnosis and treatment of victims of envenomation; (iii) the structure for emergency care, antivenom administration and prompt treatment of adverse reactions, and (iv) the existence of a tertiary care facility within a short distance from the healthcare unit. Hospitals located in municipal headquarters are at an advantage since they easily meet these criteria. However this leaves the primary care services furthest from urban centers deprived of the specific treatment for snakebites. This distortion in the supply of key medical supplies becomes more serious due to the risk of clinical complications and deaths being greater when patient travel time to a tertiary medical facility is greater than six hours, since time is crucial in achieving a favorable prognosis [5].

From the late 1980s to the early 1990s, with the construction and implementation of the Unified Health System, the acquisition of antivenoms was centralized by the Ministry of Health and distribution was no longer controlled by states and municipalities [28]. This change ensured that most of municipalities in the Brazilian Amazon region started receiving regular amounts of antivenoms. However, planning for the acquisition and distribution antivenoms is based on the number of vials used and the number of patients registered in the surveillance system. Thus, patients who do not reach a referral healthcare unit, as is commonly the case, do not figure in the official statistics. This is the situation for a great part of the populations living in indigenous reserves, revealing an inequality in access to antivenom treatment.

Despite the advances in the healthcare system, antivenom availability and accessibility are not uniform, particularly among the most vulnerable sections of the populations that inhabit remote areas in the Brazilian Amazon. Rather than being distributed to rural and indigenous healthcare facilities, where most basic health problems are managed locally by a nurse, antivenom treatment is only available in the urban areas, where physicians are present. The official Brazilian Ministry of Health guide for snakebite treatment recommends antivenom therapy be carried out under medical supervision in a hospital setting. Studies from the 1990s justified this recommendation by pointing out that the frequency of early anaphylactic reactions to the antivenoms was above 25% [29,30], thus requiring immediate access to a well-equipped hospital. Based on this older notion of the risks associated with administering antivenom, the indigenous communities deep in the Amazon are denied timely access to the antivenom. As a result, there is a significant association between indigenous status and case fatality from snakebites; about five times greater in comparison to nonindigenous people [6]. Regulation of Brazil’s pharmaceutical sector by the National Health Surveillance Agency (ANVISA), through the publication of the current Good Manufacturing Practice (GMP) standards, has led the antivenom manufacturers to upgrade their production facilities to higher standards. Over time, these products have gradually and significantly improved notably in their purity. This higher quality and purity in the antivenom product results in a lower risk of adverse events. Interestingly more recent studies conducted in the Brazilian Amazon observed that early adverse reactions to snake antivenom occur at a frequency of about 15%, and that these events were predominantly mild skin reactions [31]. Thus, making antivenom treatment available to strategically located health facilities would be a beneficial intervention and would increase the Amerindians’ access to treatment rather than exposing them to risks associated with the use of antivenom without advanced medical support.

Better transport and storage logistics of liquid antivenoms is an additional issue that needs to be addressed so that realistic and comprehensive health programs for indigenous groups can be effectively structured. These improvements would reduce the high morbidity and mortality rates associated with snakebite envenomations [3] for these vulnerable communities. Lack of antivenoms in indigenous territories is partly due to their physical-chemical characteristics, since they require storage and transport in a cold chain, as well as the risks associated with the administration of proteins derived from animals.

**Figure 4:**
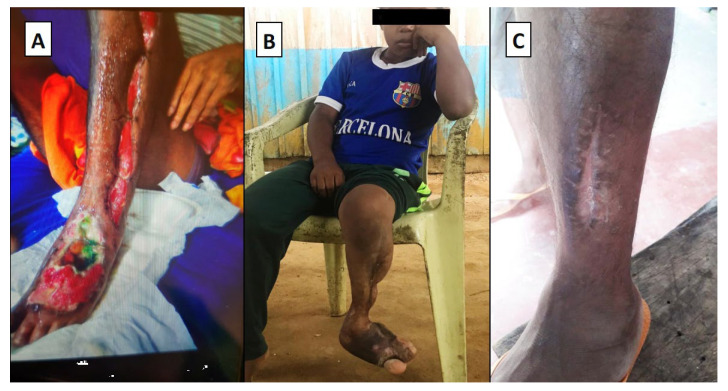
(**A**) A 19-year-old male patient from the Mura tribe who suffered a pit viper bite in the rural area of the municipality of Careiro da Várzea. Specialized care at a tertiary health unit in Manaus was only possible 10 h after the bite. During his 38-day stay at the hospital, the patient received 10 vials of antivenom. A diagnosis of compartment syndrome led to fasciotomy. The patient developed necrosis on the dorsum of the foot as seen in the image recorded on day 15 after the bite. (**B**) Patient from the Kubeo tribe; a 12-year-old male who suffered a snakebite at the age of five years while accompanying a family member to carry out agricultural activities in the municipality of São Gabriel da Cachoeira. Treated with traditional medicine as the first choice, he sought specialized care 15 h after the bite. He suffered great tissue loss, and as he grew, major motor deficits occurred. Currently, he suffers from the development of scoliosis and posture deviation. (**C**) Scars from fasciotomy on a 40-year-old male Tikuna patient, living in the rural area of Tabatinga. He was bitten by a pit viper during agricultural activities one year before the picture was taken. He received antivenom 6 h after the bite and developed compartment syndrome. Currently, the patient has difficulty walking.

These factors have contributed to the development and use of a model for the distribution and use of antivenoms. This model, unfortunately, restricts the use of the antivenom to emergency rooms and hospitals, thereby placing the indigenous peoples at a disadvantage in regards to timely access and proper application of antivenom treatment. The result of which has left them to opt for traditional therapies that are readily accessible to them. In addition, this inadequacy in the system continuously promotes under-reporting of cases, sequelae and deaths from snake envenomations among the indigenous communities. Figure 4 shows severe effects of snake envenomation from three separate cases, all characterized by severe impairment of the affected limb in which it was necessary to transport the patient to a health unit. A common feature in these cases is the consequences of delayed treatment.

Currently, five types of snake antivenom are available in Brazil: *Bothrops* AV (main antivenom), *Crotalus* AV, *Bothrops-Crotalus* AV, *Bothrops-Lachesis* AV, and *Micrurus* AV. All are produced by three national laboratories (Instituto Butantan, Fundação Ezequiel Dias, and Instituto Vital Brazil) and are provided free of cost to patients [4].

## 5. Perspectives for Decentralizing Antivenom for Indigenous Populations in the Amazon

In the 1980s, the reorganization of official antivenom manufacturing laboratories and the overhaul of the distribution network at strategic points for the application of antivenoms showed satisfactory results. However, these initiatives have remained ineffective in solving the problems faced by the most remote areas, which are where large numbers of riverine and indigenous people live [3]. Greater decentralization in the distribution of antivenoms requires complementary improvements in technological advances, logistics, and human resources capable of providing correct and prompt administration of snake antivenoms. From a technological point of view, the challenge is the large-scale production of antivenoms that do not require cold storage. Methods such as lyophilization can be adopted to produce antivenoms that can be stored at room temperature without losing their efficacy or potency. Technically, lyophilizing represents a critical step in the production of antivenom, as the operation requires a freezing protocol and drying cycles of the samples that are adjustable depending on the type of antivenom produced [32]. Huge investments are necessary in these state laboratories in order to convert at least part of their production plant to produce lyophilized products that may be used in the Amazon. The decision to initiate the production of lyophilized antivenoms in Brazil on an industrial scale depends not only on the producers’ initiative but also on the Ministry of Health, as in the 1980s. The Ministry needs to coordinate a strategic program to allocate resources to develop lyophilized products that not only retain their physicochemical stability even in high ambient temperatures but that can be safely transported over long distances.

Prioritization in the access to antivenoms depends not only on the availability of products, but also on the capacity of the professionals that will administer them. Since this is a medical intervention administered intravenously, whose risk of adverse reactions is inherent due to the antivenom’s equine-derived nature, successful administration depends on the presence of specially-trained health professionals. The health professionals need specialized training to recognize snakebite envenomation accurately, prescribe the appropriate dose of the specific antivenom, administer the antivenom correctly, and treat allergic reactions if they arise. In remote areas, the scarcity of infrastructure and medications and the difficulty in relocating quickly to a more specialized medical center makes the use of antivenoms outside the hospital environment a challenge that needs novel solutions, strategies and partnerships.

In recent years, with the improvement in the quality of antivenoms produced in the country and the pressing need to reach indigenous populations living in remote areas, it has become necessary to review the current system of using a referral services network for antivenom administration. The possibility of administration of antivenoms by paramedics and emergency medical teams as part of primary care services would undoubtedly make it possible to reduce the burden of snakebite severity among the indigenous people who, understanding the benefit of antivenom, have sought to solve the problems afflicting their community, regardless of their causes.

Decentralization of antivenom therapy is an excellent place to start. A crucial step in decentralizing the antivenom therapy service is the expansion and validation of standard operational procedures for the diagnosis and treatment of snakebites in the low complexity health units, which, with proper training, can be carried out by nurses. In other words, they need to be trained to identify severe snake envenomation symptoms, apply the appropriate antivenom, in the right dosage. In methodological terms, six implementation steps are essential: (i) development and validation of standardized operational procedures, (ii) professional training in the validated protocol at a referral health unit, (iii) implementation of a pilot study in a hospital environment at the referral unit, (iv) implementation of the protocol in primary healthcare units, v) qualitative assessment of the validated protocol: perceptions and acceptability, and (vi) postimplementation evaluation of the protocol’s effectiveness.

### 5.1. Development and Validation of Standardized Operational Procedures

This process requires that: (i) operational procedures should be explained to nurses based on the official Brazilian Ministry of Health’s guidelines [33], with adaptations that do not compromise the quality of care given to the patient; (ii) the adaptations of content should be validated using a technique, such as the Delphi Technique [34], in which the proposed instrument is judged by experts, who will express their judgment on each of the proposed items in the protocol, and; (iii) the evaluation is based on semantic issues, in which, the presentation sequence, withdrawal, addition, or modifications in each item will be questioned [35].

### 5.2. Training of Professionals in the Validated Protocol

Professional training should occur on two levels; in an initial theoretical face-to-face module and a practical module. This step should ideally occur in a tertiary reference service center. In the theoretical module, professionals would receive instructions regarding the physiopathology of snake envenomation, local and systemic clinical manifestations, instructions on the adequate storage and preparation of antivenoms, application of antivenoms and management of early reactions. In the practical module, the professional would monitor the admission, syndromic diagnosis of envenomation, classification of case severity and definition of the antivenom to be given (type and number of vials), administration of antivenom, identification, and treatment of early adverse reactions, and monitor the reversal of signs and symptoms of envenomation, and be familiar with the criteria for discharge and transfer of a patient to a reference unit.

### 5.3. Implementation of the Protocol in the Health Base Pole, at the DSEIs

In the indigenous village, the Basic Health unit must be evaluated based on epidemiological criteria (the population it would serve and the number of envenomations known to occur in the area). These units should:

(a) be staffed at all times with health care professionals compatible with the demand for care;
(b) perform and offer 24 h care;
(c) have an ambulance available for immediate transfer of patients to referral units when needed (whether due to worsening of clinical conditions or adverse reactions to antivenom treatment);
(d) have professionals trained in emergency clinical care procedures (as required by the cases);
(e) have the capacity for the proper storage of antivenoms and other drugs to be used in the patient’s care.

According to validated procedures, delivery of antivenoms to primary care units should be guaranteed with storage and transport conditions. It should be noted that ideally the professionals to be trained already work in these healthcare units within these locations and have previous experience in indigenous health matters. The Basic Health units, on the other hand, are already part of the cold chain for the distribution of medicines and immunobiologicals and have appropriate refrigeration equipment similar to the ones used to store vaccines.

### 5.4. Qualitative Assessment of the Validated Protocol: Perceptions and Acceptability

The execution phase stages should include assessments of the perceptions, acceptability, and difficulties encountered in implementing the validated protocol. The interviewed professionals’ views and experiences should compose a qualitative cross-sectional study with comprehensive concepts through textual analysis of the transcripts [36]. A semistructured interview guide should be developed with 15–20 open-ended questions, allowing the interviewer to investigate the professionals’ perceptions regarding the following: (1) clinical manifestations of snakebites; (2) difficulties in the diagnosis and determination of the antivenom dosage; (3) difficulties in discharge and referral for treatment in referral units; and (4) uncertainties during the entire patient management flow.

### 5.5. Estimate the Effectiveness of Protocol Implementation

The following indicators are essential for obtaining feedback on the application of the protocol in centers chosen for this intervention, compared to historical case series in the same area:

(a) correct diagnosis of the presence and type of envenomation, based on epidemiology, causative agent, signs and symptoms;
(b) time between diagnosis and administration of antivenom;
(c) correct classification of snakebite severity;
(d) correct type of antivenom and the correct dose to be delivered;
(e) rapid identification of the appearance of early adverse reactions and evaluation of the actions taken;
(f) assessment of the real need to transfer cases to the referral unit;
(g) evaluation of the reclassification of the severity of the snakebite and the administration of an additional dose of antivenom;
(h) evaluation of the application of the discharge criteria established in the protocol;
(i) time and completion of the notification form.

At the end of the study, the degree of agreement between the trained health professional’s transfer decision and the definition of the study’s specialist doctors should be evaluated, based on the medical records reviewed.

## 6. Final Remarks

Indigenous people in Brazil, particularly those in the Amazon region, constitute populations that are principally vulnerable to the risk of complications from snakebites, since they are generally the farthest from health care centers where antivenom is available. The current structuring of indigenous health care is disconnected from the official snakebite surveillance system and the antivenom distribution networks. This system continually contributes to the persistent underreporting of envenomation cases within indigenous communities and territories. This underreporting, in turn, contributes to delays in treatment with antivenom and probably contributes to chronic disabilities since it presents inaccurate data to national entities tasked with overseeing the distribution of vital antivenoms to health facilities. Thus, the snake antivenom is embedded in a universe of diverse social, natural, and supernatural depictions. Its use does not constitute opposition to traditional medicine, and it is often seen as an essential complementary treatment, whose accessibility represents not only more than a desire, but a right of these traditional people. The information presented here also serves as a basis for advocacy to support and promote the rights of the indigenous patients in the health care arena, and helps build the capacity to make antivenom available in the villages, as well as develops health policy initiatives focused on evidence-based care and management of snakebite patients.
